# High performing hospitals: a qualitative systematic review of associated factors and practical strategies for improvement

**DOI:** 10.1186/s12913-015-0879-z

**Published:** 2015-06-24

**Authors:** Natalie Taylor, Robyn Clay-Williams, Emily Hogden, Jeffrey Braithwaite, Oliver Groene

**Affiliations:** Centre for Healthcare Resilience and Implementation Science, Australian Institute of Health Innovation, Faculty of Medicine and Health Sciences, Macquarie University, Level 6, 75 Talavera Road, North Ryde, Sydney, NSW 2109 Australia; Faculty of Public Health and Policy, London School of Hygiene & Tropical Medicine, 15-17 Tavistock Place, London, WC1H 9SH UK

**Keywords:** High performing hospitals, Qualitative research, Improvement strategies, Systematic review

## Abstract

**Background:**

High performing hospitals attain excellence across multiple measures of performance and multiple departments. Studying high performing hospitals can be valuable if factors associated with high performance can be identified and applied. Factors leading to high performance are complex and an exclusive quantitative approach may fail to identify richly descriptive or relevant contextual factors. The objective of this study was to undertake a systematic review of qualitative literature to identify methods used to identify high performing hospitals, the factors associated with high performers, and practical strategies for improvement.

**Methods:**

Methods used to collect and summarise the evidence contributing to this review followed the ‘enhancing transparency in reporting the synthesis of qualitative research’ protocol. Peer reviewed studies were identified through Medline, Embase and Cinahl (Jan 2000-Feb 2014) using specified key words, subject terms, and medical subject headings. Eligible studies required the use of a quantitative method to identify high performing hospitals, and qualitative methods or tools to identify factors associated with high performing hospitals or hospital departments. Title, abstract, and full text screening was undertaken by four reviewers, and inter-rater reliability statistics were calculated for each review phase. Risk of bias was assessed. Following data extraction, thematic syntheses identified contextual factors important for explaining success. Practical strategies for achieving high performance were then mapped against the identified themes.

**Results:**

A total of 19 studies from a possible 11,428 were included in the review. A range of process, output, outcome and other indicators were used to identify high performing hospitals. Seven themes representing factors associated with high performance (and 25 sub-themes) emerged from the thematic syntheses: *positive organisational culture*, *senior management support*, *effective performance monitoring*, *building and maintaining a proficient workforce*, *effective leaders across the organisation*, *expertise-driven practice*, and *interdisciplinary teamwork*. Fifty six practical strategies for achieving high performance were catalogued.

**Conclusions:**

This review provides insights into methods used to identify high performing hospitals, and yields ideas about the factors important for success. It highlights the need to advance approaches for understanding what constitutes high performance and how to harness factors associated with high performance.

**Electronic supplementary material:**

The online version of this article (doi:10.1186/s12913-015-0879-z) contains supplementary material, which is available to authorized users.

## Background

High performing hospitals consistently attain excellence across multiple measures of performance, and multiple departments. Hospital performance assessment has become a key feature among many health systems in high-income countries [1], and increasingly so in low- and middle-income countries [2, 3]. Data used for such assessments have become more robust over the years and indicate often substantive variation in hospital performance, both in terms of adherence to evidence-based process of care measures and of risk-adjusted outcomes of care [4–6]. Two particular concerns have emerged in the last decade from research on hospital performance. First, hospitals are persistently lagging behind in incorporating well-established scientific knowledge into their work routines and processes—an artefact labelled as a ‘translation gap’ [7, 8]. Second, hospitals frequently fail to excel on multiple performance domains; they may achieve excellent results on some performance indicators such as in organisational structure [9], but perform below standard on others [10–12].

There is a longstanding interest in studying high performing organisations in management science [13], driven by their need as businesses to compete against multiple targets, such as price, quality and service. Statistical analysis of the associations between hospital performance rankings and hospital characteristics has received particular attention [14]. Such research is useful for identifying quantifiable relationships, but it fails to capture the wider underlying explanatory factors for high performance. It is often limited in scope and concentrates on selected indicators only [12, 15]. This focus on specific measures detracts from the larger issue that performance varies substantially not only between, but also within hospitals [16]. This has implications for those managing, contracting and regulating hospital services.

Studying high performing hospitals can be valuable if factors leading to or associated with performance can be identified and lessons learned are transferable to other hospitals. Factors contributing to high performance are likely to be complex and the wide ranges of variables that determine high performance are unlikely to be untangled by correlational analysis. Thus, the aim of this study was to assess research addressing the wider question of performance, and to generate a rich picture of the factors related to high performance in hospitals. Our specific objectives were to (i) systematically identify comprehensive evaluations of the factors related to high hospital performance, (ii) describe the methodological approaches used to identify and study high performance, (iii) create a rich picture of high performing hospitals by analysing the themes emerging from these studies, and (iv) demonstrate how the qualitative factors associated with high performance align with existing evidence.

## Methods

We gathered and assessed the evidence for high performing organisations, and synthesised the explanatory factors associated with high performance derived from qualitative research. Methods used to collect and summarise the evidence contributing to this review followed the ‘enhancing transparency in reporting the synthesis of qualitative research’ (ENTREQ) protocol [17], a completed version of which can be found in Additional file 1.

### Search strategy

We undertook a search for peer reviewed, English language studies using Medline, Embase and Cinahl between 1^st^ January 2000 and 21^st^ February 2014 following consultation with a university librarian with database and search strategy expertise (Additional file 2). We specified key words, subject terms, and medical subject headings [18] relating to: 1) the setting—hospitals; 2) methodological approach for assessing performance—quantitative; and 3) methodological approach for understanding performance—qualitative. Boolean operators and truncated terms were used to maximise the sensitivity and efficiency of the search strategy. We checked the sensitivity of the search strategy by confirming it was comprehensive enough to recognise five key papers meeting the inclusion criteria that were identified by the team during the conceptual stage of the review. Search results were combined and duplicates excluded, and the remaining citations were subject to title and abstract screening by four reviewers (NT, OG, RCW, EH). One percent (*n* = 80) of the resulting articles were double-reviewed to assess the comprehensiveness of data extraction and interpretation between coders. Following this, the remaining titles and abstracts were reviewed and kappa scores were used to assess inter-rater reliability on 5 % (*n* = 400) of titles and abstracts. Prior to the full text review, a pilot assessment was undertaken by all reviewers of 4 % of included studies, discrepancies were resolved, and changes were made to the data extraction form. The full text review was performed by three reviewers (NT, RCW, EH) on retained studies. Differences were resolved by consensus.

### Eligibility criteria

We included empirical studies that identified high performing hospitals, and used qualitative methods to examine the factors associated with high performance. Eligible studies required: 1) the use of a specific method to identify high performing organisations, 2) the inclusion of the development, testing, or use of methods or tools to identify factors associated with high performing hospital or hospital departments, and 3) an attempt to identify the factors associated with high performing hospitals or hospital departments using qualitative methods with healthcare professionals, managers, patients, patient relatives, or carers. Studies testing an intervention were eligible providing one of the additional aims was to identify factors associated with high performance. Studies were excluded if they were not peer reviewed, were descriptions of personal experiences or expert opinions, presented results of high performing organisations without investigating factors associated with high performance, presented results only relating to low performance, or barriers to high performance, or were not hospitals (i.e., were other types of healthcare organisations such as general practitioner surgeries or community clinics). Studies which included a mixture of different types of organisations including hospitals were only included if results for the factors associated with high performance were distinguished for the hospital cohort of the sample.

### Data collection process

Data from included articles were recorded in a locally developed data extraction form by three reviewers (NT, RCW, EH), and independently validated by one reviewer (OG). Data items collected were: a) the full reference, b) country, c) period of data collection, d) study type, e) study aims, f) theoretical paradigm, g) data: *n* of organisations, data types and sources used to identify high performers, methodological approach used to identify high performers, *n* of high performing organisations identified, h) methods: methods used to study context or success factors associated with high performance i) findings: context or success factors important for explaining high performance, referenced findings to theoretical paradigm, j) practical strategies, and k) implications.

### Risk of bias

We assessed the risk of bias using criteria developed by Hawker and colleagues [19], which has been used in a range of reviews. Their critical appraisal tool allows for the methodological rigour of each empirical study to be assessed through assigning ratings (very poor, poor, fair, good) across nine categories: abstract and title, introduction and aims, method and data, sampling, data analysis, ethics and bias, findings/results, transferability/generalizability, implications and usefulness (Table 1) [19]. The Hawker Tool was included in a full text practice review by three reviewers, who discussed and resolved any disagreement about usage, performed the quality assessment on all included studies, and together clarified minor uncertainties at the end of the process.

**Table 1:** Methodological rigour and risk of bias

Study	Abstract and title	Introduction and aims	Method and data	Sampling	Data analysis	Ethics and bias	Findings/results	Transferability/generalizability	Implications and usefulness
Bradley et al. (2006) [10]	Fair	Good	Fair	Good	Good	Good	Good	Good	Poor
Mannion et al. (2005) [44]	Fair	Good	Fair	Fair	Good	Very poor	Fair	Fair	Fair
Sautter et al. (2007) [37]	Fair	Fair	Fair	Fair	Poor	Poor	Fair	Fair	Fair
Cherlin et al. (2013) [32]	Good	Good	Fair	Fair	Good	Poor	Good	Fair	Fair
Landman et al. (2013) [32]	Fair	Good	Fair	Fair	Good	Poor	Fair	Poor	Fair
Rangachari (2008) [31]	Fair	Fair	Fair	Fair	Fair	Poor	Fair	Poor	Fair
Baumann et al. (2007) [47]	Fair	Fair	Poor	Poor	Poor	Fair	Fair	Fair	Poor
Rose et al. (2012) [45]	Fair	Fair	Good	Fair	Fair	Very poor	Fair	Fair	Fair
Keroack et al. (2007) [34]	Fair	Good	Good	Fair	Good	Good	Fair	Good	Good
Hockey and Bates (2010) [42]	Fair	Poor	Poor	Fair	Poor	Poor	Fair	Poor	Fair
Adelman (2012) [41]	Poor	Good	Fair	Good	Good	Poor	Fair	Fair	Good
VanDeusen Lukas et al. (2010) [36]	Fair	Fair	Fair	Poor	Fair	Poor	Fair	Fair	Fair
Kramer et al. (2008) [40]	Fair	Poor	Very Poor	Poor	Very Poor	Fair	Very Poor	Very Poor	Poor
Parsons and Cornett (2011) [35]	Fair	Fair	Fair	Poor	Fair	Poor	Fair	Poor	Poor
Curry et al. (2011) [38]	Good	Fair	Good	Good	Good	Fair	Good	Good	Fair
Puoane et al. (2008) [33]	Good	Fair	Fair	Fair	Good	Good	Good	Fair	Good
Stanger et al. (2012) [43]	Fair	Good	Fair	Good	Fair	Very Poor	Fair	Fair	Good
Kane et al. (2009) [109]	Poor	Fair	Very Poor	Fair	Very Poor	Poor	Poor	Poor	Poor
Olson et al. (2011) [48]	Fair	Fair	Fair	Poor	Good	Fair	Fair	Fair	Fair

### Syntheses of results

Key findings about the methods used to identify high performing hospitals were categorised according to measure type (outcome, process, output, other), measure specification (e.g., mortality, adverse events), level (organisation or ward/department), and data source (e.g., hospital standardised mortality ratio, accreditation or certification rating system). Contextual factors important for explaining success were analysed based on Thomas and Harden’s description of ‘thematic synthesis’ [20]. This involves identifying key themes in published studies, then going beyond the original studies to identify similarities and differences, and to propose novel interpretations, ‘lines of argument’, or ‘third-order’ concepts not found in any single study [21, 22]. An inductive approach was used, whereby initial concepts were identified, revised and added to as subsequent studies were coded. The coding was conducted by one reviewer (NT), who returned to the full text for each paper to cross-check that all the relevant data had been extracted, and generated the initial list of themes and subthemes against the supporting evidence. NT, RCW, and EH discussed concepts and quotes, and refined the themes and sub-themes as a group. The value of soft systems methodology over grounded theory methodology has been advocated [23]. Therefore, a ‘rich picture’ [24–27] was also created to provide a diagrammatical representation of how the emerging themes co-exist within a high performing organisation. Whilst there is no formal technique for the production of rich picture diagrams [28], it is recommended that base constructs are identified and the interrelationships between stakeholders are represented. Their actions or concerns and the outcome of actions or events are needed to convey a reflexive representation of the situation [29]. Information extracted from studies regarding the practical strategies that can be used for achieving high performance were then mapped against the identified themes and characteristics as a way of indicating how each strategy might be used to improve specific aspects of performance. The resulting mapping table was reviewed along with supporting quotations from each included study, to confirm assessments and achieve consensus in the approach taken to matching themes and characteristics to strategies. An amendment was made, and further work was undertaken to fine-tune the representation of the model and improve the usability of this resource. The themes, subthemes, rich picture, and practical strategies mapping results were subjected to a member checking exercise with 15 senior management and frontline staff from a large nearby teaching hospital who were interested in high performance in healthcare (in the week of 7-14^th^ July, 2014).

In a final phase, triangulation of qualitative findings with existing quantitative evidence pertaining to factors associated with high performing organisations was undertaken. One author (NT) used the theme and sub-theme word lists to systematically search for supporting literature, reviewed through approximately 90 additional papers, and mapped relevant evidence to the theme and sub-theme lists.

## Results

### Search strategy

**Fig. 1:**
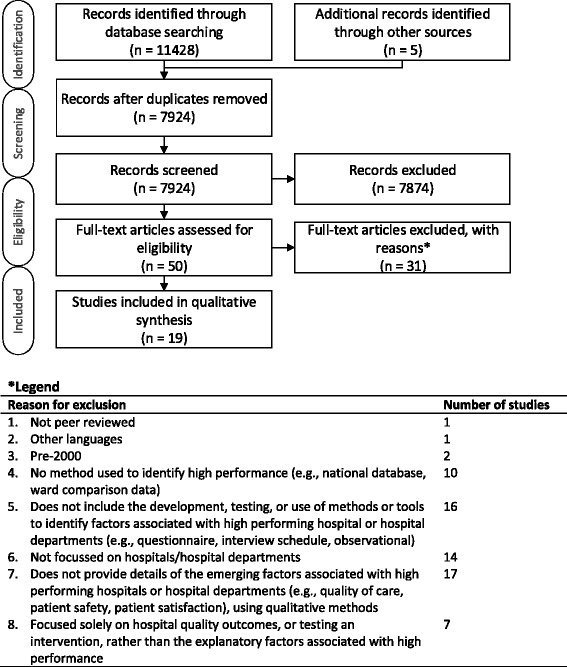
Search and review strategy (PRISMA flow diagram)

Figure 1 presents the results of the search and review strategy, utilising the Preferred Reporting Items for Systematic Reviews and Meta-analyses (PRISMA) flow diagram [30]. To summarise, the search produced 11,428 articles, which included the five papers previously identified by the team that met the inclusion criteria. Following removal of duplicates (*n* = 3504), 7924 studies were included in the title and abstract review. Agreement >85 % (kappa = 0.63) was found for pilot double coding on 1 % (*n* = 80) of titles and abstracts against the criteria, so the remaining were reviewed, and additional double coding of 5 % of all titles and abstracts (*n* = 400) produced over 98 % agreement (kappa = 0.80). Prior to the full text review, a pilot review was undertaken by all reviewers on 4 % of included studies, discrepancies were resolved, and changes were made to the review form (e.g., addition of coding for: ‘theoretical paradigm’, ‘practical strategies’, and ‘implications’, and amendments to the approach to coding the methods used to identify high performers). The full text review was performed by three reviewers (NT, RCW, EH) on 50 studies, and 19 studies were retained. Reasons for exclusion are provided in Fig. 1.

### Description of included studies

Table 2 presents the characteristics of the 19 studies included in the review. Of the total, 15 were conducted in the United States of America, three in the United Kingdom, and one study in South Africa. Twelve studies investigated both high performing and non-high performing sites. Within each study, the size of the sample frame varied considerably (range = 4 to 960 sites; *Median* = 15.5, *Inter Quartile Range* 11*–*78.25). One study did not provide the sample frame. Five studies did not identify the total number of sites that were identified as high performers, and of the 14 studies that did, the number of high performing sites identified ranged from 2 to 36 (*M* = 7, *IQR* = 5.25-14.5). The number of high performing sites investigated in each study ranged from 2 to 15 (*M* = 6, *IQR* = 3–7). Eleven studies provided a description of the location of high performing sites (e.g., regional, urban, suburban, or rural), nine studies provided information about teaching or academic status, and four studies included information about profit status and bed size.

**Table 2:** Characteristics of the included studies

Study	Country	N sites in sample frame	N sites identified as HPS	N HPS investigated	Characteristics of high performing sites
Bradley et al. (2006) [10]	USA	151	35	11	- 111-870 beds
					- 3 teaching, 8 non-teaching
					- Located in 5 regions
Mannion et al. (2005) [44]	UK	6^a^	4a	2	- NS
Sautter et al. (2007) [37]	USA	54	NS	2	- 1 suburban and 1 urban site
					- Large teaching hospitals
Cherlin et al. (2013) [32]	USA	11^a^	7^a^	7	- 6 teaching sites and 1 non-teaching site
					- Located in 4 regions
					- All non-profit
Landman et al. (2013) [32]	USA	11^a^	7^a^	7	- 317-855 beds
					- 6 teaching sites, 1 non-teaching site
					- Located in 4 regions
Rangachari (2008) [31]	USA	4^a^	2^a^	2	- 2 large teaching hospitals
					- Located in Manhattan
Baumann et al. (2007) [47]	UK	6^a^	6^a^	6	- 1 unitary authority
					- 2 shire counties
					- 1 London borough
					- 2 metropolitan boroughs
Rose et al. (2012) [45]	USA	100	13^a^	3	- Anticoagulation clinics in Veterans Health Administration sites
Keroack et al. (2007) [34]	USA	79	5	3	- NS
Hockey and Bates (2010) [42]	USA	NS	NS	3	- 2 academic medical centers
					- 1 community hospitals
Adelman (2012) [41]	USA	16^a^	16^a^	4	- 3 regional sites
					- 1 community site
					- All not-for-profit
					- 2080 -14,000 employees
VanDeusen Lukas et al. (2010) [36]	USA	7	4	4	- Medical centres in one network in the Department of Veterans Affairs
Kramer et al. (2008) [40]	USA	76	Not identified^a^	8	- 3 academic sites
					- 5 community sites
					- Located in 8 regions
					- 2 in medium sized cities
					- 3 in large cities
					- 3 in very large cities
Parsons and Cornett (2011) [35]	USA	15^a^	15^a^	15	- Not-for-profit hospitals
					- Located in 7 regions
					- 130–1000 beds
Curry et al. (2011) [38]	USA	11^a^	7^a^	7	- 6 teaching sites
					- 1 non-teaching site
					- Located in 4 regions
					- 317-855 beds
Puoane et al. (2008) [33]	South Africa	11	NS^a^	2	- Remote hospitals
					- District hospitals
Stanger et al. (2012) [43]	UK	277	NS	7	- NS
Kane et al. (2009) [109]	USA	71	36	6	- Non-profit
					- Non-teaching
					- Non-rural
					- Acute hospitals
Olson et al. (2011) [48]	USA	960^a^	10^a^	7	- NS

HPS = high performing sites

NS = information not stated in paper

^a^Clarification sought and provided by corresponding author via email

### Risk of bias

The methodological rigour of studies was assessed to indicate the risk of bias. Most of the studies were rated as ‘good’ or ‘fair’ for methods, data analysis, and results, except for four studies in which the method was not clearly explained, five studies in which the description of the data analysis was not sufficiently rigorous, and two studies which did not present enough detail in the results (Table 1).

### Methods used to identify and investigate high performers

Table 3 summarises the methods used to identify high performing organisations. Six studies used process measures (e.g., achieving a median door-to-balloon time of ≤90 min; extent of change in left ventricular ejection fraction assessment achieved over the three-year period) to identity sites as high performers. Three studies used output measures (e.g., rankings of risk adjusted anticoagulation control; blood wastage as a percentage of issue indicator), eight studies used outcome measures (e.g., internal medicine outcome measures such as rates of pneumonia and congestive heart failure; risk-standardised mortality rate, i.e., how many people per thousand die per year adjusted for hospital case mix) and six studies used other measures (e.g., a rating or scoring system, such as the UK’s National Health Service’s (NHS) star ratings based on clinical and managerial effectiveness) to rank or assess hospital performance. A combination of methods was used in two studies.

**Table 3:** Approaches taken to identify high performance

Study	Data sources	Measure type	Methodological/statistical approach used to identify high performing sites
Bradley et al. (2006) [10]	National Registry of Myocardial Infarction	Process	Hospitals who treated patients with ST-segment-elevated myocardial infarction undergoing *percutaneous coronary intervention (PCI)* were selected (*n* = 151). Within this group hospitals with median door-to-balloon times of ≤90 min for their most recent 50 PCI cases were selected (*n* = 35). All 35 hospitals were approached for interview. Interviews occurred until thematic saturation (after 11 hospitals).
Mannion et al. (2005) [44]	NHS Star ratings	Other (rating)	Hospitals were identified using the NHS star rating. Four low (0 or 1 star) and 2 high (3 star) performing hospitals were included.
Sautter et al. (2007) [37]	Blue Cross Blue Shield of Michigan Participating Hospitals Program	Process	All participating hospitals (*n* = 54) were categorised by the extent of change in left ventricular ejection fraction (LVEF) assessment achieved over a three-year period. There were four groups: Always optimal, Improved to optimal, Improved, and Not improved. 10 hospitals in total were sampled, covering all four categories and varying in size, teaching status and geography.
Cherlin et al. (2013) [32]	Centers for Medicare & Medicaid Services (CMS) Hospital Compare website	Outcome	US hospitals were selected as high or low performers if their 30-day risk standardized mortality rates were in the top or bottom 5 %, respectively, for two consecutive years. Within the top 5 % hospitals were ranked best to worst performers and in the bottom 5 % they were ranked worst to best. The hospitals were asked to participate one at a time until theoretical saturation (reached after 14 hospitals). For top performers only those that remained in the top 5 % for a third consecutive year were retained (*n* = 7)
Landman et al. (2013) [32]	CMS Hospital Compare Web	Outcome	30-day risk-standardized mortality rates were calculated by dividing the hospital’s predicted number of deaths by the expected number of deaths within 30 days of admission. Hospitals were eligible for inclusion as high or low performers if their risk-standardized mortality rate was in the top 5 % or bottom 5 % of performance for 2 consecutive years.
Rangachari (2008) [31]	New York State hospital administrative database	Process	Hospitals were categorised as good and poor performers using the percentage of uncertain coding (0-5 % = good, 95 % - 100 % = poor). A purposeful sample of two good and two poor performers were selected from those willing to participate in the study.
Baumann et al. (2007) [47]	NS	Outcome, Other (reporting, rating)	A multistage process was used: First, statistical model was used to shortlist authorities. The model used a range of data from 1998 to 2000 to predict rates of discharge delay from acute hospitals. The authors examined rates of delays and emergency readmissions data for these sites over a longer period (1998–2002) to ensure sustained high performance. These results were cross referenced with joint review reports by health and social care inspectorates. Star ratings and delayed discharge performance data for hospitals and primary care trusts within the short-listed authorities were examined to ensure good performance. Hospitals selected as high performers ensured a mix of geography and local authority type.
Rose et al. (2012) [45]	Veterans Administration (details cited in Rose 2010 [110]and Rose 2011 [111])	Output	Rankings of risk adjusted anticoagulation control at Veterans Administration sites were used to identify the best 10 and worst 10 sites. Three of the top 10 and three of the bottom 10 were selected, representing six different states.
Keroack et al. (2007) [34]	University HealthSystem Consortium, an alliance of 97 university teaching hospitals	Process, Output, Outcome, Other (equity score)	A composite index of patient-level process and outcomes data, including indicators on preventable complications and mortality rates, evidence based practices and equity of care, was calculated from discharge abstract data from 79 Academic Medical Centers. Six institutions (three top and three average performers) were selected for site visits, covering different geographical areas and levels of hospital ownership.
Hockey and Bates (2010) [42]	Hospital Quality Alliance program	Outcome	Hospital Quality Alliance (HQA) data were used to identify hospitals with consistent performance during the preceding two years from the top (high performing) and bottom deciles (low performing) on internal medicine outcome measures (pneumonia and congestive heart failure). Twelve hospitals were consistent performers (either high or low) and all were asked to participate in the study. Five hospitals agreed to participate (3 high performers and 2 low performers).
Adelman (2012) [41]	Malcolm Baldrige National Quality Award (MBNQA) or state-level Baldrige award	Other (award recipient)	Hospitals which had won either a MBNQA or state level Balridge award in the last 7 years were the target sample. Two MBNQA and two Balridge recipients participated.
VanDeusen Lukas et al. (2010) [36]	Veterans Administration (VA) Network	Process	Of 3 interested VA networks, a single network was chosen, which included 7 sites. An organisation model positing that implementation of EBPs is enhanced by the presence of three critical organizational components (active top leadership commitment; links to senior management structures and processes; multi-disciplinary evidence-based clinical process redesign) was implemented in all 7 sites. Hand-hygiene compliance scores and the overall fidelity of the model was calculated for each site. Site with a score over 3 were considered high fidelity (*n* = 4).
Kramer et al. (2008) [40]	The National Magnet Hospital Profile	Essentials of Magnetism (EOM) score	The EOM scale (available for 76 EOM tested hospitals) was used to identify the highest and second highest scoring hospitals in each region of the country (8 regions in total). A balanced sample of highest and second highest scoring hospital was taken reflecting hospital type (community/academic). To select local units within the hospital, staff nurse experience, certification, and education of local units were correlated with that of the sample; the unit was included if differences in the three sets of correlations were not significant. In addition to adequate Registered Nurse representation, staff nurses’ reported Control over Nursing Practice (CNP) scores had to be above the hospital mean for the unit to be included.
Parsons and Cornett (2011) [35]	American Nurses Association Magnet Recognition Program	Other (rating)	A convenience sample of Chief Nursing Officers was selected to be interviewed from the whole pool of magnet recognised hospitals that were willing to participate (*n* = 15).
Curry et al. (2011) [38]	Centers for Medicare & Medicaid Services Hospital Compare	Outcome	Hospitals that ranked in the top 5 % of performance on risk-standardized mortality rates for acute myocardial infarction care during both years were eligible for inclusion. Selection continued until theoretical saturation, which occurred after 7 high performing hospitals.
Puoane et al. (2008) [33]	Unclear	Outcome	11 hospitals were given the same intervention to improve care of malnourished children. Two high performing and two poorly performing hospitals were purposively selected based on their improvement or lack of improvement in case-fatality rates.
Stanger et al. (2012) [43]	Blood Stocks Management Scheme	Output	Historical data on the inventory management of blood stock from 277 hospitals was used to create an indicator on blood waste (wasted as a percentage of issue (WAPI)). Seven units with low WAPI percentage were selected for further analysis.
Kane et al. (2009) [109]	Data provided by the hospitals to the state (regulatory requirements)	Outcome	Researchers calculated the most recent available five consecutive years of operating margin performance, including annual performance and the average annual trend. Only non-teaching, non-profit, acute care hospitals were included in the sampling frame. Hospitals were stratified by local market areas and ranked on operating margin. 36 top performing hospitals were selected based on these rankings and 6 agreed to participate.
Olson et al. (2011) [48]	American Heart Association (AHA) /American Stroke Association	Process	Top-performing sites were defined as those in the top 1 % of all hospitals contributing to the “Get with the Guidelines – Stroke” programme (*n* = 1315) for achieving a door-to-needle time of less than 60 min. Hospitals administering tPA to fewer than 12 patients (average of less than one patient per month) were excluded (*n* = 960). All hospitals who were asked to participate agreed. 13 personnel in total at 7 top performing hospitals were interviewed.

NS = information not stated in paper

Table 4 presents the qualitative methods used to identify factors associated with high performance. Mixed methods (quantitative and qualitative methods) approaches to identifying high performing organisations were applied in 12 studies, and seven studies employed qualitative methods only. All included studies used interviews to identify factors of high performance. Nine studies also undertook a site visit or observation, six studies performed an additional document review, and three studies included other methods. Five studies did not state how many participants were interviewed specifically in sites identified as high performers. Of those that did, the range of participants interviewed in each was 12–906 (*Median* = 34.5, *IQR* = 15.25-64.75). Where the information was provided, professions of participants interviewed included physicians (k = 12), nurses (k = 9), administrators (k = 5), clinical staff (k = 4), chief executive or board member (k = 4), chief medical officer or medical director (k = 3), chief nursing officer or nursing director (k = 4).

**Table 4:** Methods used to identify factors associated with high performance

Study	Data collection	Participant numbers and sampling method	Participant professions (N)
Bradley et al. (2006) [10]	Interviews	Interviews	Interviews
	- Open-ended interviews conducted with individuals and groups, lasting 1–1.5 h	- Total participants: 122	- Physicians (23), Nurses (37), Administrators (44), Quality management staff (8), Clinical/support staff (10)
		- Participants per site: approximately 10-12	
		- Participants at HP sites: 122	
		- Sampling: Nonprobability	
Mannion et al. (2005) [44]	Interview	Interviews	Interviews
	- Semi-structured	- Total participants: Approximately 60^a^	- Chief Executive and Medical Director
	Document review	- Participants per site: 8-11	
	- Commission for Health Improvement report and internal documents	- Participants at HP sites: 40^a^	
	Site Visits	- Sampling: NS	
Sautter et al. (2007) [37]	Interviews	Interviews	Interviews
	- 1.5 h	- Total participants, Participants per site and Participants at HP sites: NS	- Senior hospital leadership, chief cardiologist and Medical chief of staff
	- Semi-structured	- Sampling: Nonprobability	- Physician (6), Nurse (26), Administration (21), Clinical staff (4)
		- Total participants: 57	
	- Conducted with individuals and groups	- Participants per site: NS	
		- Participants at HP sites: 38^a^	
		- Sampling: Nonprobability	
Cherlin et al. (2013) [32]	Interviews	Interviews	Interviews
	- Standard discussion guide used for 1 h interview		
	Site visits		
	- 1-2 day visits to review previous years’ activities.		
Landman et al. (2013) [32]	Interviews	Interviews	Interviews
	- Standard interview guide used	- Total participants: 85	- Physician (*n* = 17), Nurse (*n* = 29), Administration (*n* = 32), Clinical staff (*n* = 7)
	Site visits	- Participants per site: NS	
	- Emergency Medical Services medical director during EMS training session (1 site).	- Participants at HP sites: 46^a^	
		- Sampling: Nonprobability	
Rangachari (2008) [31]	Interview	Interviews	Interviews
	- Semi-structured interview conducted with individuals and groups, lasting 0.75-1 h	- Total participants: Approximately 24^a^	- Hospital administrators^a^, Physician leaders^a^, Coding managers^a^
	Survey		
	- Online survey on knowledge exchange related to quality measurement	- Participants per site: Approximately 6^a^	
		- Participants at HP sites: Approximately 12^a^	
		- Sampling method: NS	
		Survey	
		- Total participants: 65	
		- Participants per site: NS	
		- Participants at HP sites: 45	
		- Sampling: Nonprobability	
Baumann et al. (2007) [47]	Interviews	Interviews	Interviews
	- Topic guide	- Total participants: 42	- Directors of nursing, Service managers, Social services team managers, Lead hospital discharge coordinators, Care managers, Discharge facilitators
		- Participants per site: (hospitals/acute trusts) 14^a^	
		- Participants at HP sites: 14^a^	
		- Sampling: Nonprobability	
Rose et al. (2012) [45]	Interviews	Interviews	Interviews
	- Semi-structured (lasting 0.3-1 h)	- Total participants: 56	- Direct-care ACC staff (25), ACC support staff (6), Pharmacy administration (11), Physicians (6), Other staff (7), Supervisor of clerks (1)
	Observations	- Participants per site: NS	
	- Approximately 4 h of clinical care.	- Participants at HP sites: 31^a^	
	Documents	- Sampling: Nonprobability	
	- ACC-related documents		
Keroack et al. (2007) [34]	Documents	Interviews	Interviews
	- Internal documents	- Total participants: NS	- CEO, Governing board members, Chief medical officer, Chief nursing officer, Chief financial officer, Clinical department chairs, Division chiefs, Nursing unit directors, Medical unit directors, Residents, Medical and nursing staff
	Site visits	- Participants per site: “dozens”	
	- Document verification and information gathering (interviews) during 1.5 days	- Participants at HP sites: NS	
	Interviews	- Sampling: NS	
	- Open ended questions		
Hockey and Bates (2010) [42]	Interviews	Interviews	Interviews
	- Semi-structured, 0.75-1 h interview	- Total participants: 17	- Senior residents (2), Senior internists (3), Cardiologist (1), Pulmonologist (1), Nephrologist (1), Hospitalists (4), General internists (5)
		- Participants per site: NS	
		- Participants at HP sites: NS	
		- Sampling: Nonprobability	
Adelman (2012) [41]	Document review	Interviews	Interviews
	- MBNQA/Baldrige award application.	- Total participants: 20	- CEO, Baldrige lead for the hospital, Clinical services area director, Frontline manager/clinical services area supervisor, Frontline nurse.
	Interviews	- Participants per site: 5	
	- Semi-structured	- Participants at HP sites: 20	
		- Sampling: NS	
VanDeusen Lukas et al. (2010) [36]	Interviews	Interviews	Interviews
	- Semi-structured	- Total participants: 137^a^	- Director, Chief of staff, Nurse executive, Associate director, Clinical redesign team members, Clinical redesign team leaders, Project improvement advisor, Front-line staff
		- Participants per site: NS	
		- Participants at HP sites: 71^a^	
		- Sampling: NS	
Kramer et al. (2008) [40]	Interviews (three types)	Interviews	Interviews
	- 2-h, group orientation interviews	- Total participants: 906^a^	- Nurse manager, Director group, Staff nurses, Nurse managers, Physicians, Chief nursing officer, Chief operating officer, Departmental representatives
	- Individual interviews	- Participants per site: NS	
	- Semi-structured expert interviews	- Participants at HP sites: 906^a^	
	Observations	- Sampling: NS/Nonprobability	
	- 26 council meetings and lunch meetings (6 hospitals)		
	Operational and evaluation data		
	- Internal documents and CWEQII tool		
Parsons and Cornett (2011) [35]	Interviews	Interviews	Interviews
	- 1 h	- Total participants: 15	- Chief Nurse Officers (15)
		- Participants per site: 1^a^	
		- Participants at HP sites: 15	
		- Sampling: Nonprobability	
Curry et al. (2011) [38]	Interviews	Interviews	Interviews
	- 1 h	- Total participants: 158	- Physicians (19), Nurses (52), Administration (65), Clinical staff (22)
	- Standard discussion guide used	- Participants per site: 14 (average)	
	Site visits	- Participants at HP sites: 109^a^	
	- Debriefing sessions (organizational psychologist and site visit teams)	- Sampling: NS	
Puoane et al. (2008) [33]	Observations	Interviews	Interviews
	- Structured approach to observing ward procedures during 3 days per hospital	- Total participants: 32^a^	- Matrons, Hospital superintendent, Doctors, Sister-in-charge or her deputy, Professional and enrolled nurses
	Interviews	- Participants per site: 8^a^	Focus groups
	- Semi-structured interviews (0.75-1 h)	- Participants at HP sites: 16^a^	- Nurses (various categories)
	Focus groups	- Sampling method: NS	
	Quantitative measures	Focus groups	
	- Hospital environment data	- Approx 8 participants per focus group^a^	
		- Recruitment: NS	
Stanger et al. (2012) [43]	Interviews	Interviews	Interviews
	- 1 h	- Total participants: NS (7 interviews)	- Transfusion laboratory managers
	- Open-ended	- Participants per site: NS	
		- Participants at HP sites: NS (7 interviews)	
		- Sampling: Nonprobability	
Kane et al. (2009) [109]	Interviews	Interviews	Interviews
	- 0.75-2 h	- Total participants: NS (73 interviews)	Board members and CEOs
	- Semi-structured	- Participants per site: 5–7 (unclear)	
		- Participants at HP sites: NS (44 interviews)	
		- Sampling: Nonprobability	
Olson et al. (2011) [48]	Interviews	Interviews	Interviews
	- Semi-structured	- Total participants: 13	- Stroke coordinator, Stroke manager, Neurologist, Radiologist, ED nurse manager, Nurse manager, Pharmacist, Physician
		- Participants per site: 1-4	
		- Participants at HP sites: 13	
		- Sampling: Nonprobability	

HP = high performing

NS = information not stated in paper

^a^Clarification sought and provided by corresponding author via email

### Themes representing high performing organisations

**Fig. 2:**
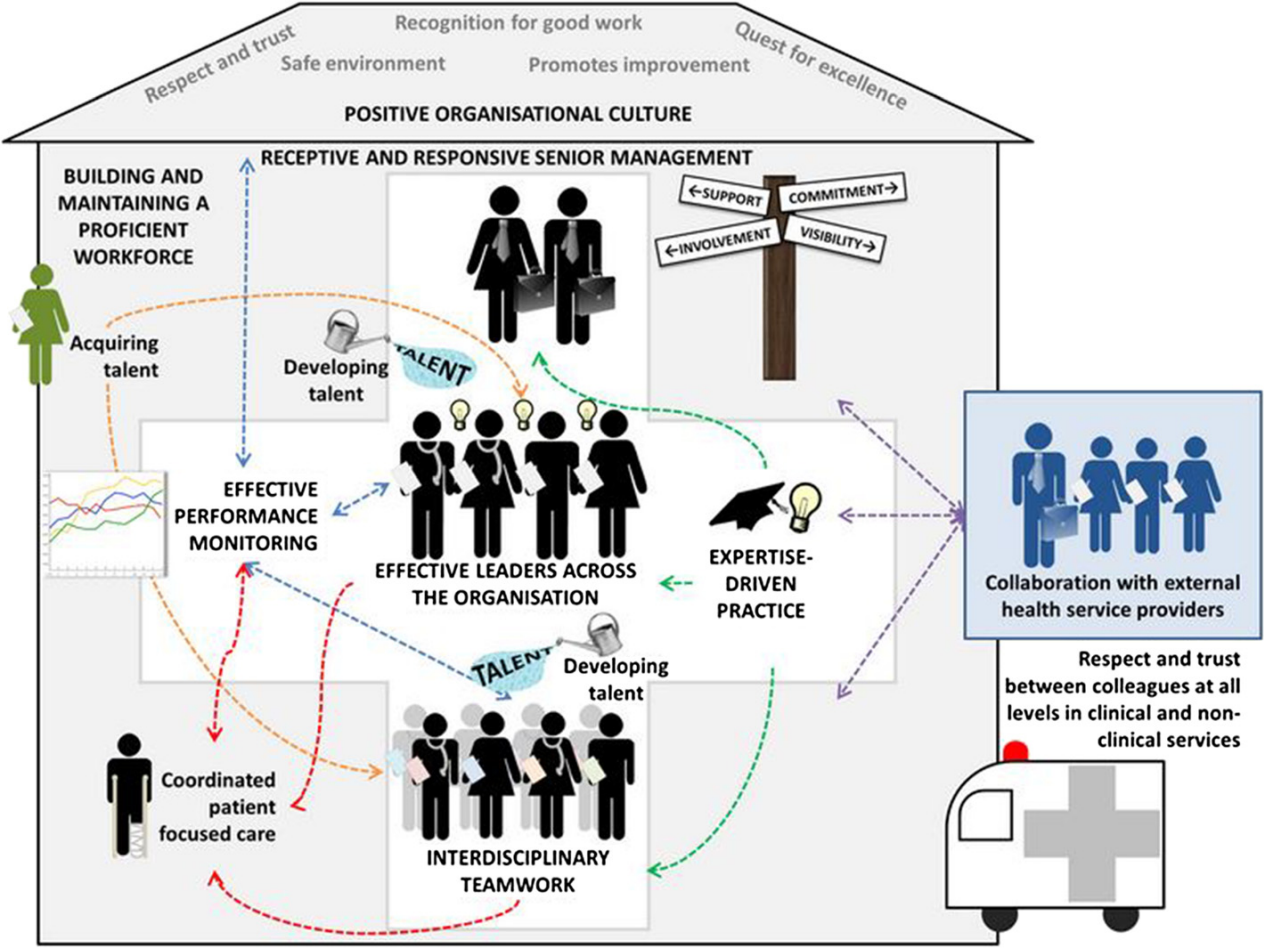
Rich picture of high performing hospitals

Seven themes representing key factors integral to high performing hospital organisations emerged from the thematic syntheses: *positive organisational culture*, *senior management support*, *effective performance monitoring*, *building and maintaining a proficient workforce*, *effective leaders across the organisation*, *expertise-driven practice*, and *interdisciplinary teamwork*. These factors, alongside the associated characteristics (sub-themes), are summarised in Table 5 and described with reference to supporting evidence below. The interplay of these factors and characteristics within an organisational context is represented in Fig. 2.

**Table 5:** Themes and sub-themes representing factors associated with high performing organisations

Theme	Subtheme	Number of strategies
1) Positive organisational culture	1a) Respect and trust between colleagues at all levels in clinical and non-clinical services [30, 29, 8, 31]	16
	1b) Relentless quest and unwavering focus for excellence [32–35]	13
	1c) Recognition and compensation for good work [36, 32, 38, 35]	8
	1d) Safe, non-threatening environment [39, 34]	17
	1e) Promotes values for improvement [40, 39]	17
2) Receptive and responsive senior management	2a) Support [29, 8, 39, 32]	24
	2b) Involvement [31, 32, 29]	5
	2c) Access and visibility [40, 39]	8
	2d) Commitment [33, 36, 40]	10
3) Effective performance monitoring	3a) Accurate measurement and goal setting [8, 32, 36, 41]	18
	3b) Sophisticated data systems [35, 42, 43, 33, 41]	6
	3c) Using data for continuous feedback and improvement [35, 8, 30, 45, 40, 38, 36, 31, 46]	28
	3d) Accountability [42, 40, 32, 36]	8
4) Building and maintaining a proficient workforce	4a) Acquiring and developing talent [42, 29, 43, 40, 39, 38, 33]	9
	4b) Aligning staff with the organisational vision [42, 29, 32, 36]	13
	4c) Effective dissemination of policy and processes [30, 31, 41, 46]	6
	4d) Mandatory and specialised training [30, 40, 38, 41, 31]	11
5) Effective leaders across the organisation	5a) Commitment and responsibility [8, 33, 36, 31]	5
	5b) Support staff to enhance performance [8, 31, 46]	24
	5c) Mutual respect [8, 31]	11
6) Expertise-driven practice	6a) Frontline autonomy and flexibility based on experience and expertise [8, 35, 32, 41]	18
	6b) Trust and empowerment for innovation and creativity [40, 33, 32, 36]	20
7) Interdisciplinary teamwork	7a) Effective multi-disciplinary and multi-level collaboration and communication [8, 30, 29, 32, 39, 34, 38, 36, 31, 46]	22
	7b) Collaboration with external health service providers [30, 45, 29]	12
	7c) Coordinated patient focused care [8, 38, 46, 40, 36, 30, 43]	13

### Theme 1: Positive organisational culture

From the literature, positive organisational culture is represented by five characteristics, including ‘respect and trust between colleagues at all levels in clinical and non-clinical services’. High performing hospitals demonstrated respect and support between clinical, non-clinical, and support staff, and that the contribution of each staff member to the delivery of care was valued [31, 32]. Studies provided evidence to suggest that levels of mutual respect pertained between colleagues, disciplines, and departments [10, 33].

A ‘relentless quest and unwavering focus for excellence’ was apparent. Studies demonstrated that high performing hospitals held the philosophy that consistent, ongoing efforts were needed for improvement in order to fulfil a desire to provide the highest level of care and maintain a reputation of excellence [34–37]. Staff from high performing sites indicated that vigilance, and an ability to ‘focus despite the noise’ was needed to successfully set and monitor priorities among competing pressures [35, 36].

‘Recognition and compensation for good work’ also contributed to a positive organisational culture. Recognition came in different forms, such as an endorsement from the senior staff, funding for high performing front line staff to implement their healthcare improvement ideas, and providing rewards to leaders to allocate to their own staff [34, 37–39]. Financial, and time-based, forms of compensation were offered, as well as logistical support, such as the provision of play areas for children adjacent to meeting sites [40].

A positive organisational culture was represented by a ‘safe, non-threatening environment’ whereby staff felt safe to speak out and take risks associated with improvement, and were encouraged to voice concerns or suggest ideas for improvement [36, 41]. High performing hospitals demonstrated the development of ‘cultures of safety’ through employee teams and initiatives in which all employees felt comfortable speaking up [39].

A positive organisational culture was also achieved through ‘promoting values for improvement’. High performing hospitals had an organisation-wide ‘mission’ or ‘vision’ which promoted a culture of continuous improvement, and that quality and safety was at the heart of the organisation [41, 42].

### Theme 2: Receptive and responsive senior management

There were four characteristics emblematic of this second theme. Senior management ‘support’ was evident in high performing organisations through examples such as an appreciation from staff of the support from senior management for encouraging clinicians to cooperate with non-clinical staff (e.g., for the implementation of documentation systems) [31], and providing resources for improvement initiatives [10].

‘Involvement’ from senior management also contributed to a high performing hospital through interaction and communication with staff, hands-on style, and proactive and continuous participation with improvement activities [31, 33, 34]. The value of senior management’s ‘access and visibility’ was evident in the identified literature. Staff highlighted the value of having senior management who were easy to speak to and who actively made themselves visible using approaches such as an open door policy, making it easier for employees to interact with them and jointly solve problems [41, 42].

‘Commitment’ was the final characteristic representing a receptive and responsive senior management. High performing hospitals demonstrated explicit, and sustained senior management commitment to high quality care, which was evident to staff within the organisation [35, 38, 42].

### Theme 3: Effective performance monitoring

The third key theme—effective performance monitoring—was represented by four characteristics, the first of which was ‘accurate measurement and goal setting’. Evidence suggested the value staff placed on explicit goals that were set based on reliable data [10]. Emphasis was placed on transparency and visibility of data [43], the need for team members to have the same goals and to understand how data are being gathered [10], and for teams to align multiple goals in order to achieve high quality patient outcomes [38].

‘Sophisticated data systems’ supported effective performance monitoring. High performing hospitals indicated that it was beneficial to invest resources in well-functioning infrastructures to monitor clinical and financial performance, and support quality improvement [37, 44]. Software packages helped staff to handle their patient loads effectively (e.g., by improving workflow and ensuring patients are not lost to follow up), improve workflow, track and cross-match patients (e.g., for identification of compatible blood donors), and to undertake internal performance measurement [43, 45].

Effective performance monitoring was also represented, chiefly by the characteristic ‘accountability’. Evidence suggested that it was important to establish clear and largely unequivocal lines of upward accountability using individual and organisational outcome data in order to be able to clearly identify and address poor performance, and to recognise and reward staff for high performance [36, 38, 42, 46].

The literature suggested that it is important to ‘use data for continuous feedback and improvement’. Establishing systems for redesigning clinical processes and providing feedback on physician performance was described as a way of maximising opportunities for physicians to reach quality targets [37]. Good quality, credible data was used at individual, team, and organisational levels to highlight problem areas (e.g., delays), motivate changes, test new methods (e.g., comparison of mattress overlays for patients with high risk of pressure ulcers), support adherence to protocols, promote success amongst peers and senior management, develop action plans, identify gaps in knowledge and skills that can be targeted through specific training, and sustain new processes over the long term [10, 32, 33, 38, 40, 42, 47, 48].

### Theme 4: Building and maintaining a proficient workforce

The fourth theme identified was represented by four characteristics. Evidence from the included studies indicated the importance of ‘acquiring and developing talent’. For example, organisations applied behavioural standards in selection and performance review, and hired staff with high qualifications [39, 45]. The benefits of retaining good staff were also emphasised by senior leaders who pointed out that as people work together longer, they become more comfortable communicating with one another [41]. Harnessing potential (e.g., through specific training and talent academies for future leaders, or identifying and mentoring champions for the acquisition of evidence-based practice) was also identified as a key factor for high performance [35, 40, 42, 44].

A human resources function was also identified; that of ‘aligning staff with the organisational vision’. High performing organisations placed priority on recruiting staff who displayed a commitment to following a corporate rather than a purely professional agenda, and made efforts to shape the values and behaviour of key staff in accordance with the organisational philosophy, norms, and culture [38, 49]. Peer interviewing, and regular discussions as part of the annual review of physician contracts are examples of approaches taken to encourage an employee’s alignment with the organisational vision [34, 39].

‘Effective dissemination of policy and processes’ was another characteristic associated with building and maintaining a proficient workforce. High performing hospitals recognised the need for predetermined, explicit patterns of care that team members – including external care providers (e.g., ambulatory services) – are aware of [32, 33, 48], and highlighted the importance of established systems during potentially vulnerable periods (e.g., staff rotations) to ensure crucial tasks are managed safely and effectively [43].

The provision of ‘mandatory and specialised training’ was also key to this theme. There was a strong focus on robust training and education initiatives in high performing hospitals, whereby investment for education and quality was emphasised through devoted hospital resources [32, 42]. Examples of training include educational programmes on evidence-based practice, nurse ‘boot camps’ [40], in-service training on the ward [33], reflective multidisciplinary team learning [32], and staff briefings to raise awareness of key standards of practice [43].

### Theme 5: Effective leaders across the organisation

Effective leadership across the organisation, with three sub-themes, was a fifth key factor. Leaders demonstrated ‘commitment and responsibility’ towards caring and quality [10, 35]. Leaders ranging from the CEO, clinical leads, directors of nursing, medicine, and administrators, felt a responsibility for their teams and were described as individuals who continuously strive to hit the mark for the best outcomes in the world [33, 38].

Leaders ‘support staff to enhance performance’ through monitoring performance, talking with individuals and teams and delivering feedback, and providing the necessary resources to improve their practice [10, 33, 48]. By sharing information about their own targets, staff members were able to see how they are contributing to the bigger picture [39].

‘Mutual respect’ was also a factor associated with high performing hospitals. This was demonstrated by evidence describing clinician leaders as typically highly respected individuals who could be persuasive with their peers, highly respected directors of nursing with strong leadership qualities, and supportive directors of medicine who are respectful of nursing staff [10, 33].

### Theme 6: Expertise-driven practice

Expertise-driven practice was a sixth factor, represented by two characteristics. ‘Frontline autonomy and flexibility based on experience and expertise’ was apparent through hospital approaches to quality improvement that widely held physician preferences for participation in the design of programmes which would affect their own work [37], and the emergence of grassroots projects without the need for pressure from central oversight committees [34]. High performing organisations enabled front line staff to continuously refine protocols based on rapid cycle feedback [10], processes which tended to evolve over a number of years using staff expertise to make small incremental changes and achieve optimal performance [43].

High performing hospitals provided employees with ‘trust and empowerment for innovation and creativity’ through the use of problem solving teams, pushing decision making towards the front line, and in policies and practices that sought to reduce status distinctions [39]. Feeling trusted by senior management enabled healthcare professionals to thrive on innovation and creativity, and persevere in trial-and-error efforts to improve through, for example, choosing to add indicators of quality to those that were already mandated [34, 38, 42].

### Theme 7: Interdisciplinary teamwork

The seventh theme associated with high performing hospitals—interdisciplinary teamwork—was represented by three characteristics. ‘Effective multi-disciplinary and multi-level collaboration and communication’ was evidenced through the use of multifaceted strategies to foster and support strong communication and coordination amongst disciplines and departments working together over time to achieve common goals [10, 32]. These kind of approaches, involving a combination of staff types and levels ranging from administrators, paramedics, technicians, nurses, clinicians, and senior management, was described as ‘an alliance’ [50], and a ‘horizontal team’ [10], which involves ‘collaboration’, ‘good teamwork’ [33], and ‘shared decision making’ [40].

High performing hospitals were effective at ‘collaborating with external health service providers’ where necessary, appropriate, or both. This was demonstrated through the recognition by hospitals of the need to keep out-of-hospital care providers informed on the latest evidence-based care for patients with specific conditions [32, 47], and the communication between hospital healthcare practitioners and administrators, and services such as general practices and social services, for effective and timely treatment of patients during each stage of their journey through the system [31, 47].

‘Coordinated patient focused care’ was demonstrated in high performing organisations through interdisciplinary teamwork, which enabled hospital staff to achieve their ultimate objective—the best outcomes for their patients. Examples of evidence-based patient focused care include recollections from staff about the use of evidence-based practice teams balanced with clinical autonomy to make decisions for the benefit of the patient [40], and specific processes with timely cooperation from a range of departments to ensure a care team is ‘ready for the patient’ [48]. Teams cultivated a shared, patient-focused mission to improve care and outcomes, and benefited from feedback on the status of their patients from both a reflective learning and motivational perspective [10, 32, 42].

### Practical strategies

Fifty six practical strategies that can be used to adopt the factors associated with high performance were identified within the 19 included studies (Table 5). The mapping exercise resulted in between 5 and 28 strategies that demonstrated the potential to contribute to attaining a range of the 26 characteristics representing the seven key factors associated with high performance. Additional file 3 (*this file will also be hosted on the AIHI website at*http://aihi.mq.edu.au/resources/practical-strategies) provides an interactive demonstration of how each characteristic associated with the seven key factors might be accomplished or improved through the use of specific strategies. For example, the strategy ‘ensure timely, bidirectional communication between the hospital and other teams/care providers’ [32] can be used to leverage ‘respect and trust between colleagues at all levels in clinical and non-clinical services’ (positive organisational culture), ‘effective multi-disciplinary and multi-level collaboration and communication’, and ‘collaboration with external health service providers’ (interdisciplinary teamwork), and the strategy ‘targets are set based on experience and are adjusted as necessary’ can be used to contribute to ‘accurate measurement and goal setting’ and ‘using data for continuous feedback and improvement’, (effective performance monitoring), ‘frontline autonomy and flexibility based on experience and expertise’ (expertise-driven practice), and ‘evidence-based patient focused care’ (interdisciplinary teamwork) [43].

### Member checking and triangulation of evidence

The study findings were presented to a group of 15 senior management and frontline healthcare professionals from a large tertiary care hospital who were interested in high performance in healthcare (in the week of 7-14^th^ July 2014). Participants provided face validity to the results by indicating that, in their experience, the themes represented the kinds of factors that would enhance performance in their own organisation, and highlighted that practical strategies for improving in each of the areas would be useful for organisations. The additional review of 90 quantitative, evidence-based papers resulted in the inclusion of evidence from 54 studies demonstrating the relationships between hospital performance and measures representing our themes/sub-themes.

## Discussion

This systematic review provides a comprehensive assessment of the published literature, identifying qualitative factors associated with high performing hospitals. We have presented the methodological approaches used to identify and study high performance, generated a rich picture of high performing hospital organisations based on emerging themes, and demonstrated how practical strategies might be used to contribute to achieving a high performing organisation.

### Factors associated with high performance

The qualitative factors associated with high performance identified in this review both align with and elaborate on some of the quantitative based evidence and broader theories of organisational performance and healthcare quality within the literature.

### Positive organisational culture and high performance

The ambiguity associated with both the definition and accurate measurement of organisational culture generates difficulties in fully understanding what constitutes culture and confirming relationships between culture and high performance [51, 52]. Our findings regarding ‘positive organisational culture’ align with *positive* representations of Schein’s [53] identifiable levels of culture. More specifically, ‘respect and trust between colleagues at all levels in clinical and non-clinical services’, and a ‘safe and non-threatening environment’ represent positive *‘assumptions’* (the unconscious, taken for granted beliefs, perceptions, thoughts and feelings), whereas ‘recognition and compensation for good work’, ‘a relentless quest for unwavering excellence’ and ‘promotes values for improvement’ represent *‘espoused values and beliefs’* (the strategies, goals, philosophies – e.g., explicit statements made by staff such as ‘we have a focused discipline with a philosophy that we are never done for clinical quality improvement’ [35]).

It is likely that many of the other characteristics associated with the remaining themes from our findings represent aspects of culture (e.g., ‘sophisticated data systems’ might align with ‘artefacts’ – visible organisational structures and processes from Schein’s model). At this point it is useful to refer to the rich picture (Fig. 2), which depicts a positive organisational culture as the ‘roof’ which covers an organisation with positive over-arching beliefs, philosophies, and actions, which infiltrate throughout the system. The rich picture also indicates that acceptance and integration of the other six factors will likely build or reinforce a positive organisational culture.

### Receptive and responsive senior management and high performance

Previous research has indicated relationships between characteristics relating to *receptive and responsive senior management* such as ‘support’, ‘involvement’, ‘commitment’, ‘access and visibility’, and positive staff perceptions and subsequent organisational performance. For example, senior hospital executives who conduct walkrounds – which include formats such as informal hallway conversations, breakroom discussions over snacks, auditorium presentations, and ‘safe tea-time’ [54], can increase employee perceptions that hospital leaders view safety as a high priority, are committed to safety, and responsive to issues identified by those on the clinical frontlines [55, 56]. Such strategies can increase possibilities for a comfortable dialogue between leaders and frontline staff to improve care processes, leading to better safety outcomes [57]. Recent evidence for the impact of senior management was provided by Schwendimann et al. [54] who surveyed 706 hospital units and found that those units with ≥60 % of healthcare professionals reporting exposure to at least 1 executive walkround had significantly higher safety climate, greater patient safety risk reduction, and a higher proportion of feedback on actions taken as a result of walkrounds compared with those units with <60 % of caregivers reporting exposure to walkrounds.

### Effective performance monitoring and high performance

Relationships between high performance and *effective performance monitoring* through ‘accurate measurement and goal setting’, ‘sophisticated data systems’, ‘using data for continuous feedback and improvement’, and ‘accountability’, are evident in the literature. For example, Mannion et al. [58] demonstrated that high performing trusts in the NHS had robust performance management and monitoring arrangements to support organisational aims, and clear and largely unequivocal lines of accountability, but there were also risks to excessive managerial approaches to measuring and monitoring [59]. Furthermore, West et al. [60] provided evidence from a study of 61 hospitals indicating that appraisals utilising goal setting were a significant predictor of reduced patient mortality. A range of evidence has demonstrated data feedback can improve practice if it is perceived as credible and valid by physicians [61–66].

### Building and maintaining a proficient workforce and high performance

Characteristics aligned with *building and maintaining a proficient workforce* have been associated with high performing organisations. The benefits of ‘aligning staff with the organisational vision’ can be seen in a study by Bart et al. [67], whereby satisfaction with a well specified organisational mission positively influenced commitment to the mission, which in turn influenced employee behaviour, and this was associated with better organisational outcomes. ‘Effective dissemination of policy and processes’ by human resource management (HRM) has been suggested as pivotal to the implementation of high performance work practices at the front line [68]. This has been highlighted by evidence linking HRM practices that increase team stability and improve teamwork among frontline employees to reductions in the average length of patient stay [69] and shorter procedure completion times [70]. With regards to ‘acquiring and developing talent’, and ‘providing mandatory and specialised training’, evidence indicates that high performing regional groupings place emphasis on recruiting and retaining staff with a high commitment to a corporate agenda [58], and that selective hiring is related to perceptions of higher quality care delivery [68]. Extensive and sophisticated training has been correlated with perceptions of higher quality care delivery [68] and lower patient mortality [60, 71]. Positive associations have also been found between training and acquisition and retention of essential employees [72, 73], perceived overall organisational performance [72, 74–77], and a clear and strong relationship between organisational support for training and subsequent performance [78].

### Effective leaders across the organisation and high performance

There is evidence for the association between *effective leaders across the organisation* and high performance in the literature. The sub-themes representing this factor (i.e., ‘commitment and responsibility’; ‘supporting staff to enhance performance’; ‘mutual respect’) are key facets of transformational leadership (i.e., encouraging new ideas from employees, attending to needs, acting as a mentor, being a good role model, and articulating vision) [79], which has demonstrated strong effects on employee and organisational outcomes [80]. For example, leaders who demonstrate ‘commitment and responsibility’ to a safety climate through personal example tend to heighten safety motivation and participation in voluntary safety activities (e.g., helping co-workers with safety-related issues and attending safety meetings) amongst subordinates [81]. Furthermore, Michie and West [82] claim that trust and respect are at the heart of good leader-follower relations, and are effective in achieving good performance [83–88].

### Expertise-driven practice and high performance

Relationships between aspects of *expertise-driven practice* identified in our review, namely ‘frontline autonomy and flexibility based on experience and expertise’ and ‘trust and empowerment for innovation and creativity’, and high performance, have been demonstrated in the literature. For example, Aiken et al. [89] provided evidence for the association between increased autonomy and decision making latitude and lower patient mortality rates, and other research has demonstrated that deference to expertise (through patient care which migrated to bedside caregivers who had more expertise with a specific patient) was associated with less deterioration in paediatric intensive care [90]. These outcomes may be due to the fact that locating expertise, autonomy and responsibility at lower hierarchical levels creates opportunities for continued individual and organisational learning [82] and a context for richer interactions that can improve information quality [91], cross-functional relationships [92], and coordination [69]. With regard to ‘trust and empowerment for innovation and creativity’, evidence from a study of over 500 NHS team indicates that teams with support for innovation and reflexivity are more effective in delivering patient care [85]. Furthermore, employee empowerment has been associated with lower patient mortality rates [89], predicts subsequent organisational productivity [93–96], and is an antecedent of quality in patient care [97, 98]. It has been suggested that employees who experience psychological empowerment feel more committed to their job, resulting in higher levels of performance [93, 99–101].

### Interdisciplinary teamwork and high performance

Interdisciplinary teamwork [102, 103], through ‘effective multi-disciplinary and multi-level collaboration and communication’, ‘collaboration with external health service providers’, and ‘coordinated patient focused care’, has also been linked to high performance. For example, interventions to increase team diversity and interdependence have led to a range of organisational outcomes, including decreased patient volume, length of stay, and hospital charges in acute inpatient and trauma team settings [104, 105], as well as increased compliance with treatment recommendations made by allied health professionals [104]. Advantages of effective communication with external health service providers have been demonstrated in the reduction of wasted visits by community staff [106]. Furthermore, coordinated care has been rated by patients as one of seven key factors that influence their perceptions of quality [107], and relational coordination (i.e., ‘coordinating work through relationships of shared goals, shared knowledge, and mutual respect’) has been associated with improved quality of care and decreased length of stay [108].

### Limitations

This review revealed large variation in the type and quality of the methods used to assess high performance. The principal methodological weaknesses identified in these studies were the use of largely invalidated instruments to assess organisational performance, and a lack of detail regarding the approaches taken to analyse qualitative data, as indicated by the ratings using the Hawker tool to assess risk of bias. Nonetheless, the themes which emerged demonstrate consistencies in the perceptions healthcare employees have about what factors are important for high performance in hospitals. Although it is not possible to make definitive conclusions about the influence of particular factors associated with high performance, we have attempted to provide triangulated evidence of these relationships from previous literature to substantiate our findings. The definition of ‘high performance’ was narrow in most studies, in that it was classified based upon a specific process (e.g., achieving a median door-to-balloon time of ≤90 min) or outcome (e.g., risk standardised mortality rate), rather than on the basis of multiple process, outcome, and output measures. This does not consider the important question from a management perspective of how to attain excellence across multiple domains of an organisation, which is an important area for future research.

Although our approach was systematic, and informed by experts in applying robust search strategies, we may have missed key words or made too little use of potentially effective medical subject headings, Boolean operators and truncated terms. However, we did attempt to validate the sensitivity of our strategy by testing for recognition of five papers the team had previously identified in the literature that met the inclusion criteria, and this was successful. The standardisation of medical subject headings and keywords in studies published within this field would aid improvements in the outcomes of literature searches for systematic review purposes.

## Conclusions

This systematic review of literature is a key step in understanding factors associated with high performing hospitals. Although the review provides an insight into some of the methods used to identify high performers, and has yielded ideas about the factors important for success, it has also emphasised the need to advance approaches for understanding what constitutes high performance and how to improve those factors associated with high performance. Nevertheless, this review moves beyond correlational analysis to disentangle some of the complexity associated with high performance, and provides insights that may be useful for both developing research hypotheses and practical strategies for improvement.

## Additional files

Additional file 1:
**Final search.** Completed ENTREQ checklist. Additional file 2:
**Final search. Search strategy for Medline, Embase and Cinahl.**
Additional file 3:
**Practical strategies.** An interactive PowerPoint Show illustrating practical strategies that can be used to achieve the factors associated with high performance. PowerPoint must be installed in order to view the file.

## Abbreviations

CEO: Chief Executive Officer
ENTREQ: Enhancing transparency in reporting the synthesis of qualitative research
HRM: Human resource management
NHS: United Kingdom’s National Health Service
PRISMA: Preferred Reporting Items for Systematic Reviews and Meta-Analyses
