# Supplementary material for: High performing hospitals: a qualitative systematic review of associated factors and practical strategies for improvement
Source: BMC Health Serv Res. 2015 Jun 24;15:244. doi: 10.1186/s12913-015-0879-z (PMC4478709; doi:10.1186/s12913-015-0879-z)
Supplement: Additional file 2: — Final search. Search strategy for Medline, Embase and Cinahl. [file 12913_2015_879_MOESM2_ESM.pdf]

Final search

1. ((hospital* adj6 rank) or (hospital* adj6 perform*) or (hospital* adj6 benchmark*) or (hospital* adj6 standard*) or (hospital* adj6 score*) or (hospital* adj6 balanced scorecard*) or (hospital* adj6 excellence) or (high* adj6 rank*) or (hospital* adj6 low*) or (hospital* adj6 high*)).tw.
2. health care surveys/ or interviews as topic/ or focus groups/ or questionnaires/ or self report/
3. case stud*.mp.
4. field notes.mp.
5. (perception* or belief*).tw.
6. (health surve* or interview* or focus group*).tw.
7. qualitative research/
8. (qualitative study* or ethnograph*).tw.
9. 2 or 3 or 4 or 5 or 6 or 7 or 8
10. 1 and 9
11. "high-perform*".tw.
12. "low-perform*".tw.
13. "top-perform*".tw.
14. "bottom-perform*".tw.
15. ((organisation* adj6 high*) or (organisation* adj6 low*) or (organization* adj6 high*) or (organization* adj6 low*)).tw.
16. ((organisation* adj6 top*) or (organisation* adj6 bottom*) or (organization* adj6 top*) or (organization* adj6 bottom*)).tw.
17. 1 or 11 or 12 or 13 or 14 or 15 or 16
18. 9 and 17
19. health*.mp.
20. 18 and 19
21. limit 20 to (english language and yr="2000 -Current")
22. A qualitative assessment of practices associated with shorter door-to-needle time for thrombolytic therapy in acute ischemic stroke.m_titl.
23. What distinguishes top-performing hospitals in acute myocardial infarction mortality rates? A qualitative study.m_titl.
24. (Cultural characteristics of 'high' and 'low' performing hospitals).m_titl.
25. (Organizational factors associated with high performance in quality and safety in academic medical centers).m_titl.
26. (Organizational characteristics of high- and low-performing anticoagulation clinics in the Veterans Health Administration).m_titl.
27. limit 18 to (english language and yr="2000 -Current")
28. 22 and 27
29. 23 and 27

30. 24 and 27
31. 25 and 27
32. 26 and 27
