# Supplementary figures and images for: High performing hospitals: a qualitative systematic review of associated factors and practical strategies for improvement
Source: BMC Health Serv Res. 2015 Jun 24;15:244. doi: 10.1186/s12913-015-0879-z (PMC4478709; doi:10.1186/s12913-015-0879-z)

## Slide 1
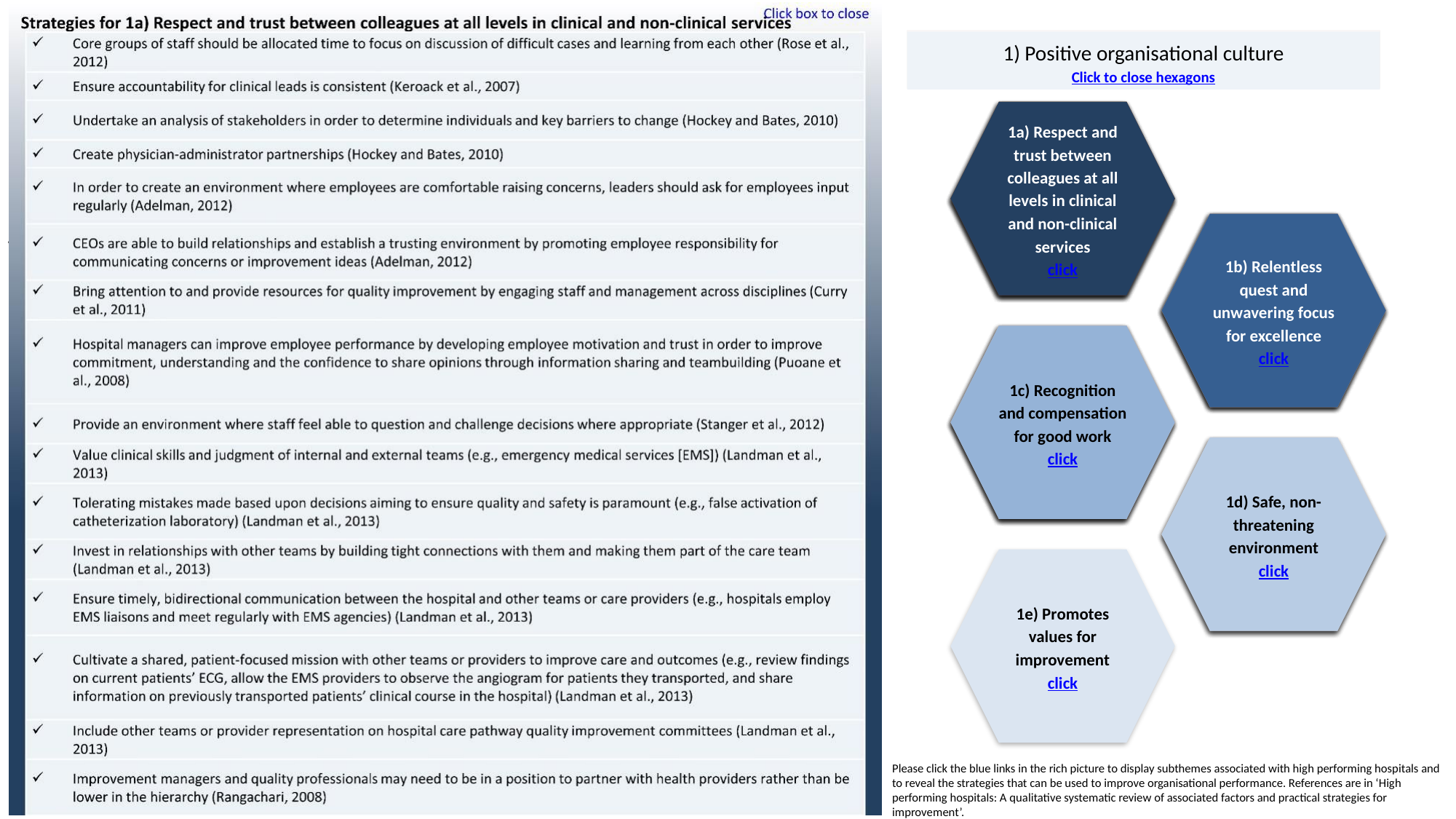

Supplement: Additional file 3: — Practical strategies. An interactive PowerPoint Show illustrating practical strategies that can be used to achieve the factors associated with high performance. PowerPoint must be installed in order to view the file. [file 12913_2015_879_MOESM3_ESM.ppsx]
